# Human leukocyte antigen haplotype phasing by allele-specific enrichment with peptide nucleic acid probes

**DOI:** 10.1002/mgg3.65

**Published:** 2014-01-19

**Authors:** Nicholas M Murphy, Colin W Pouton, Helen R Irving

**Affiliations:** 1 Drug Discovery Biology, Monash Institute of Pharmaceutical Sciences, Monash University (Parkville Campus)Melbourne, Victoria, 3052, Australia; 2 Department of Preimplantation Genetic Diagnosis, Melbourne IVF344 Victoria Parade, East Melbourne, Australia

**Keywords:** Haplotyping, HLA haplotype, HLA matching, *HLA-DRB1*, peptide nucleic acid

## Abstract

Targeted capture of large fragments of genomic DNA that enrich for human leukocyte antigen (HLA) system haplotypes has utility in haematopoietic stem cell transplantation. Current methods of HLA matching are based on inference or familial studies of inheritance; and each approach has its own inherent limitations. We have designed and tested a probe–target-extraction method for capturing specific HLA haplotypes by hybridization of peptide nucleic acid (PNA) probes to alleles of the *HLA-DRB1* gene. Short target fragments contained in plasmids were initially used to optimize the method followed by testing samples of genomic DNA from human subjects with preselected HLA haplotypes and obtained approximately 10% enrichment for the specific haplotype. When performed with high-molecular-weight genomic DNA, 99.0% versus 84.0% alignment match was obtained for the specific haplotype probed. The allele-specific target enrichment that we obtained can facilitate the elucidation of haplotypes between the 65 kb separating the *HLA-DRB1* and the *HLA-DQA1* genes, potentially spanning a total distance of at least 130 kb. Allele-specific target enrichment with PNA probes is a straightforward technique that has the capability to improve the resolution of DNA and whole genome sequencing technologies by allowing haplotyping of enriched DNA and crucially, retaining the DNA methylation profile.

## Introduction

The human leukocyte antigen (HLA) system is a region with a dense concentration of highly polymorphic genes involved in immune function, characterized by local sequence conservation within haplotype groupings (Horton et al. 2008; Lehne et al. 2011). Due to the extensive number of multigenic autoimmune genetic disease associations that the HLA system also carries, improving the resolution of sequencing platforms is a priority (Stewart et al. 2004). The *HLA-DRB1* (MIM #142857) gene locus exhibits extreme polymorphism with 1166 unique alleles reported to date (Robinson et al. 2003). The phased DNA sequence is of particular importance in haplotype matching in unrelated haematopoietic stem cell transplantation (Fuchs et al. 2010).

An aim commonly encountered in genomics is to phase genetic sequences along a chromosome efficiently, economically and with reliability (Tewhey et al. 2011). Determining haplotypes from mutations and alleles is problematic when the genetic distance between polymorphisms extends farther than the polymerase chain reaction (PCR) can reliably amplify (Crawford and Nickerson 2005; The International HapMap 3 Consortium 2010). Studies of multigenic disorders are confounded by traditional linkage analysis using familial pedigree, and in the context of full genome sequencing, reliable and economical methods for complete chromosome phasing are at present technically demanding (Botstein and Risch 2003). Sequence-specific target enrichment has potential in sequencing applications, to improve signal resolution, decrease costs, and increase the power of genome-wide association studies or haplotype/disease association studies (Baker 2010; Mamanova et al. 2010). Next-generation DNA sequencing technologies frequently employ target-capture techniques to achieve sequence-specific target enrichment, for example, in exome sequencing (Ng et al. 2009; Clark et al. 2011). Significantly increasing the length of the captured target DNA has the capacity to increase the power of whole genome sequencing (Peters et al. 2012) by, for example, including distal *cis*-regulatory genomic elements that do not fall within the protein coding regions. Phasing can be enhanced by the incorporation of long read sequencing (Voskoboynik et al. 2013), however, for repetitive intergenic regions greater than 10 kb, traditional tools must supplement genome assembly.

Peptide nucleic acids (PNAs) are a class of synthetically produced single-stranded nucleic acid probes characterized by exchange of the deoxyribose phosphate backbone of DNA for an achiral uncharged polyamide backbone (Nielsen et al. 1991). PNAs exhibit a range of unique properties; they have high DNA-binding affinity, thermal stability, can invade double-stranded DNA, and are resistant to both protease and nuclease degradation. Given the significance of the HLA region and the importance of stratifying the role of HLA alleles in disease, we investigated a method for purifying DNA samples by targeting with a sequence-specific biotinylated PNA (Demidov et al. 1994; Zhang et al. 2000). Our aim was to develop a simple DNA capture method by targeting alleles of a gene at the *HLA-DRB1* locus using PNA probes. The HLA-DRB1*01 and HLA-DRB1*03 alleles were targeted with biotinylated PNA (biotin-PNA) probes and enriched with neutravidin-coated plates. Initially, we modeled enrichment of the template of genomic DNA by constructing two plasmids, each containing a unique allele-specific insert of the second exon of the *HLA-DRB1* genes. We used PNA probes with or without biotinylation, designed to target a specific 22 bp sequence in the second exon of the HLA-DRB1*01 allele and a specific 16 bp sequence in the second exon of the HLA-DRB1*03 allele. The enrichment procedure was then adapted for enrichment using genomic DNA that had been extracted using a method to produce high-molecular-weight DNA (100–240 kb), targeting the *HLA-DRB1* locus on chromosome 6.

Our results here demonstrate PNA capture of the high-molecular-weight genomic DNA with the HLA-DRB1*03 allele enriched for the HLA-DRB1*03, HLA-DQB1*02 (MIM #604305), HLA-DQA1*05 (MIM #146880) haplotype. Significantly, this allele is implicated directly in autoimmune disorders such as celiac disease (Louka et al. 2002) and Sjögren's syndrome (Cruz-Tapias et al. 2012) or by association with multiple sclerosis (Luckey et al. 2011), systemic lupus erythematosus (Relle and Schwarting 2012), and type 1 diabetes (Bluestone et al. 2010).

## Materials and Methods

### PNA probes

PNAs were synthesized by Panagene Inc. (Daejeon, South Korea), and made up in a solution of 10:2:1 dimethyl formaldehyde:H_2_O:trifluoroacetic acid. Two nonbiotinylated PNA probes were used as negative-binding controls, and two biotinylated PNAs as positive controls to establish the protocol for each of the HLA-DRB1*01 (GenBank: X88793.1) and HLA-DRB1*03 (GenBank: JQ804938.1) plasmid constructs (Table 1). The HLA-DRB1*01-specific PNAs were coupled covalently at the N-terminus to Alexa Fluor 488 (Molecular Probes, Invitrogen, Grand Island, NY) and the HLA-DRB1*03-specific PNAs were coupled to Alexa Fluor 532 in order to optimize the enrichment parameters of plate binding and washing.

**Table 1:** Sequence of PNAs designed to target the HLA-DRB1*01 allele and HLA-DRB1*03

PNA Target	Sequence
[Alexa Fluor 488]-PNA*01	TG TGG CAG CTT AAG TTT GAA TG-Lys-AlexaFluor488
[Alexa Fluor 488]-PNA*01-Biotin	Biotin-OOO-E-TGT GGC AGC TTA AGT TTG AAT G-E-Lys-AlexaFluor488
[Alexa Fluor 532]-PNA*03	Lys-GAG TAC TCT ACG TCT G-Lys-AlexaFluor532
[Alexa Fluor 532]-PNA*03-Biotin	Biotin-OOO-GAG TAC TCT ACG TCT G-Lys-AlexaFluor532

“T,” “G,” “C,” “A” each denote standard PNA bases, “O” denotes a spacer, “Lys” denotes a lysine residue, and “E” the standard Panagene Inc. PNA linker.

### Plasmid enrichment

Construction of the two plasmids is described in Data S1. For PNA hybridization, approximately 4.0 *μ*g of each plasmid DNA was incubated with 40 pmol of PNA. Plasmid constructs were hybridized to either perfectly matched (PM) or mismatched (MM) fluorescently labeled PNAs. Each reaction was carried out in a PCR tube in 10 mmol/L sodium phosphate buffer (pH 7.5) with 1 mmol/L EDTA in a total volume of 100 *μ*L. Samples were hybridized using a modification in the method of Braasch and Corey (2001) involving 10° decrements; 95°C for 10 min, 85°C for 5 min, 75°C for 5 min, 65°C for 10 min, 55°C for 5 min, 45°C for 5 min, 35°C for 10 min, and 4°C for 10 min. Samples were then placed on a preblocked Reacti-Bind Neutravidin Coated 96-Well Plate (Pierce, Rockford, IL) that had been washed three times with Tris-buffered saline (25 mmol/L Tris, pH 7.2 and 150 mmol/L NaCl). Samples were incubated overnight at 4°C on a horizontal rocker at 50 rpm, and were then washed seven times with Tris-buffered saline. Each sample of wash solution was assayed for fluorescence using an EnVision 2101 microplate-reader (PerkinElmer, Melbourne, Australia) to determine when unbound PNA had been washed off (Data S2). The instrument was calibrated to detect fluorescence of Alexa Fluor 488 using a 488/8 nm excitation filter and a 520/8 nm emission filter, and in a separate assay for Alexa Fluor 532 labeled PNAs using a 530/8 nm excitation filter and 560/10 nm emission filter. The bound PNA:DNA product was removed from the plate by aspirating with boiling water three times into a 1.5 mL microcentrifuge tube and subsequently precipitated using sodium acetate before being resuspended in 20 *μ*L dH_2_O.

### Haplotyping assay using genomic DNA

The method used above to enrich for target plasmids was later tested using genomic DNA and 15 pmol of the [Alexa Fluor 532]-PNA*03-Biotin probe, with nonprobe samples as controls. The probe concentration was reduced in order to adapt the assay to the increased molecular weight of the genomic DNA. This decreased concentration was the threshold of probe/target ratio that could be used, while retaining a sufficient level of fluorescence so that each assay wash could be monitored. High-molecular-weight genomic DNA was extracted from a HLA-DRB1*01,03 blood sample obtained from the Australian Bone Marrow Donor Registry (ABMDR) via a modified protocol. The Wizard Genomic DNA Extraction Kit (Promega) protocol was used with the following modifications. During the protein precipitation step, samples were rotated at less than 100 rpm, and the protein component was not precipitated. Samples were centrifuged at 500*g*. The supernatant was added to 70% isopropanol to precipitate any DNA remaining in solution, with mild rotation on a vertical rocker for 5 min. DNA was then collected via a glass rod, using a gentle circular motion and added to pure DNA was determined using pulse field gel electrophoresis (PFGE). Aliquots of DNA samples were mixed with low-melting point agarose (Bio-Rad, Sydney, Australia), and prepared for PFGE by slicing each agarose block into quarters. The 0.8% agarose gel in ×0.5 TBE was run at 14°C for 24 h on a CHEF PFGE System (Bio-Rad), rotating 120° at 6 V/cm, every 60 sec. Samples were run against both a *Saccharomyces cerevisiae* DNA Ladder (New England Biolabs, Ipswich, MA) and *Saccharomyces pombe* DNA ladder (BioRad). The gel was visualized following immersion in ethidium bromide and ×0.5 TBE (Birren et al. 1988; Herschleb et al. 2007).

Haplotype-specific enrichment of genomic DNA was investigated using a similar method to that described for plasmids, with the following modifications: PNA/DNA hybridization was performed in phosphate-buffered saline with 10 mmol/L EDTA using a simplified modification in the Braasch and Corey (2001) method by heating to 95°C, followed by cooling to room temperature for 10 min, prior to overnight incubation at 4°C on a horizontal rocker. Samples were exposed to only two washes in order to preserve the integrity of the DNA bound to the microplate. Unbound material was aspirated by placing the microplate at an angle of 45° and applying slow aspiration away from the plate well-bottom. This was followed by immediate addition of the wash buffer, making several rotations around the upper edge of the microplate with Tris-buffered saline and one wash with H_2_O both at room temperature. DNA from duplicates was pooled to improve qPCR resolution. The separated products were amplified with allele-specific primers for *HLA-DRB1* (I1-RB1, I1-RB2 for HLA-DRB1*01 and I1-RB9, I2-RB28 for HLA-DRB1*03; Kotsch et al. 1999) for qPCR quantitation, and with generic *HLA-DQA1* primers for sequencing (Scharf et al. 1986). For the amplification of the *HLA-DQA1* gene locus, a set of generic primers was selected from the literature to amplify a 242 bp region of the *HLA-DQA1* gene (Scharf et al. 1986). qPCR was performed as previously described with one modification; the annealing temperatures were all increased from 61 to 63°C. Electropherogram peak height ratio analysis of the *HLA-DQA1* was used to quantify relative yield of DNA. Four gDNA controls were compared to the pooled test samples. Samples were compared to the two reference sequences for the HLA-DQA1*05 (GenBank: GU014287.1) (in strong positive linkage disequilibrium with the HLA-DRB1*03 allele; Louka et al. 2003) and HLA-DQA1*01 (GenBank: HG315526.1) (in strong positive linkage disequilibrium with the HLA-DRB1*01 allele; Myhre et al. 2002). Five polymorphic bases were selected for peak height measurement and relative yield determination.

### Data analysis

Statistical analysis was performed using two-way analysis of variance (ANOVA) with Bonferroni's post hoc test, using the statistical package GraphPad Prism 5 (GraphPad Software Inc., La Jolla, CA). Results are presented as mean ± SEM and differences at *P* < 0.05 were considered significant. qPCR results were analyzed using LinRegPCR to determine copy number (Ruijter et al. 2009). The software package QSV analyzer was used to normalize samples relative to control (nonenriched DNA samples) and to calculate the relative allele ratios of polymorphic SNPs (Carr et al. 2009).

## Results

### Single-target plasmid enrichment

The yield of DNA from the haplotype enrichment was quantified using allele-specific primers (Table S1) and qPCR. The products were compared to standard curves made using the two plasmid constructs containing the second exons of HLA-DRB1*01 and HLA-DRB1*03 alleles (*R*^2^ 0.993 and 0.997). To validate the haplotype enrichment procedure, it was necessary to evaluate the extent of nonspecific binding to the neutravidin plates. A series of control experiments were performed with plasmids alone, or with plasmids complexed to nonbiotinylated probes. Initially, we used the fluorescence of the biotin-PNA probes to confirm that the probe was in solution and was able to bind to neutravidin-coated microplates (data not shown). This experiment indicated the potential for using biotin-PNAs to selectively purify DNA in mixtures.

**Figure 1:**
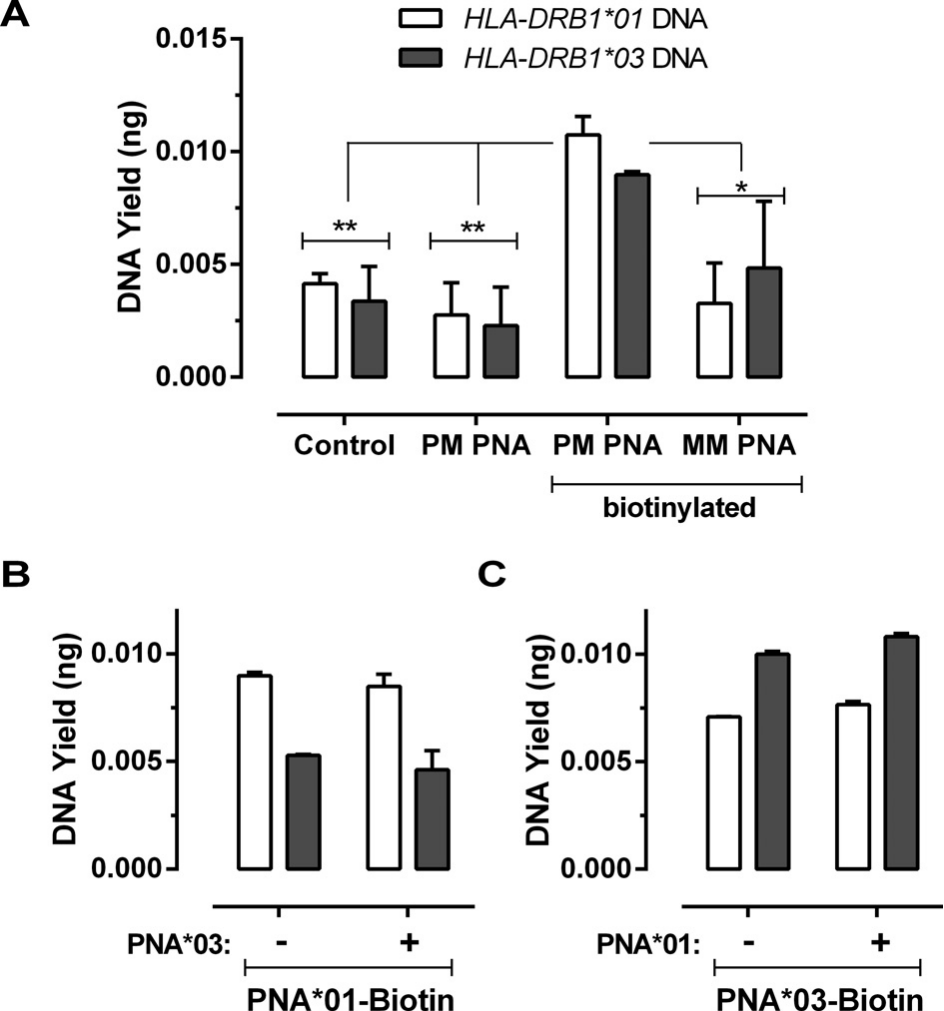
DNA yield obtained in the plasmid enrichment assay. (A) DNA yield obtained by capture of single alleles with single PNAs. (B) Plasmid DNA yield of samples modeled as pseudoheterozygotes containing equal amounts of HLA-DRB1*01 and HLA-DRB1*03 DNA, incubated with biotinylated PNA targeting the HLA-DRB1*01 DNA. (C) Biotinylated PNA targeting the HLA-DRB1*03 plasmids, either in the presence or absence of a noncompetitor nonbiotinylated mismatched (MM) PNA. Clear bars indicate the DNA yield amplified with the HLA-DRB1*01-specific primers, gray bars indicate the HLA-DRB1*03-specific primers.

Real-time qPCR was used to quantitate the number of target DNA molecules after enrichment experiments. When the plasmid enrichment assay was performed in the absence of probe approximately 4 pg of plasmid DNA was recovered after washing and subsequent aspiration of the plates with boiling water (see Materials and Methods). When plasmids were hybridized to nonbiotinylated PM probe, a marginally lower yield was recovered, though this difference was not significant. This is likely to be due to an overlapping binding site of the PNA probes to the PCR forward priming positions. When plasmid was hybridized with biotinylated PM probe, the yield from each PM probe to target was significantly higher compared to plasmid alone (*P* = 0.003; two-way ANOVA), to plasmid hybridized with nonbiotinylated PM probes (*P* = 0.004; two-way ANOVA), and to plasmid hybridized with sequence MM probe (*P* = 0.03; two-way ANOVA), indicating that the PM biotinylated probe:DNA complex had bound to the plate and the specificity of the hybridization significantly increased the recovered yield (Fig. 1A).

Two alleles of the *HLA-DRB1* gene were targeted to investigate the feasibility of conducting the enrichment of a single allele from a heterozygote. The two plasmids were then pooled in equal amounts to model pseudodiploid HLA-DRB1*01,03 heterozygote samples. Samples were probed with either the respective biotinylated PM probe alone or with a noncompetitive MM probe, which would be expected to bind weakly to the other allele, that is, the DNA sequence that was not the target for enrichment. A significantly increased yield of the targeted HLA-DRB1*01 plasmid was recovered when it was incubated with the appropriate biotinylated PM probe (*P* = 0.002; two-way ANOVA), both in the presence and absence of the MM HLA-DRB1*03 PNA probe (Fig. 1B). Similarly, a significantly increased yield of the HLA-DRB1*03 plasmid was obtained when probed with its respective PM biotinylated PNA (*P* = 0.001; two-way ANOVA), regardless of the presence of noncompetitor nonbiotinylated MM PNA (Fig. 1C). Comparison of the amplification of the samples enriched with PNA probes did not show any difference in amplification efficiency compared to nonprobe controls. The total yield of enriched DNA was lower than expected when compared to the fluorescence of bound PNA, however, this can partly be explained by the loss of product during the sodium acetate DNA precipitation and recovery steps.

### Sequencing of plasmid enrichment assay

The results obtained by sequencing the PCR-amplified products of plasmid enrichment experiments supported the hypothesis that an enrichment of the targeted allele had been achieved by binding biotin-PNA:DNA to the neutravidin plates. When the [Alexa Fluor 532]-PNA*03-Biotin probe was used to enrich for the HLA-DRB1*03 plasmid, the sequence of the PCR product had a mean alignment score of 99.5%. The alignment score against the nontarget HLA-DRB1*01 plasmid was 91.6%, reflecting the presence of expected polymorphisms characteristic of HLA-DRB1*03. In the corresponding experiment to enrich for HLA-DRB1*01 with the [Alexa Fluor 488]-PNA*01-Biotin probe, the DNA sequence had a mean alignment score of 97.7% for the HLA-DRB1*01 plasmid versus 91.5% for the HLA-DRB1*03 plasmid (Table 2). Comparative quantification of the electropherogram peak height was performed. Examples of corresponding 21 bp sequences from the enriched plasmid constructs containing the *HLA-DRB1* second exon allele inserts are shown in Table 3, indicating the highly contiguous identity of the PM-enriched targets using standard base-calling software.

**Table 2:** Sequence analysis of the two plasmid enrichment assays using mixed HLA-DRB1*01 and HLA-DRB1*03 plasmids

	HLA-DRB1*01:01:01 (reference sequence)		HLA-DRB1*03:01:01:01 (reference sequence)	
		
Probe	Sequence gaps (total)	Identities (total)	Sequence gaps (total)	Identities (total)
[Alexa Fluor 488]-PNA*01-Biotin	0	97.6% (422/432)	2	91.7% (396/432)
[Alexa Fluor 488]-PNA*01-Biotin, [Alexa Fluor 532]-PNA*03	0	97.0% (419/432)	2	92.4% (399/432)
[Alexa Fluor 532]-PNA*03-Biotin	4	91.6% (372/406)	0	99.5% (430/432)
*Probe*: [Alexa Fluor 532]-PNA*03-Biotin, [Alexa Fluor 488]-PNA*01	4	90.4% (367/406)	0	99.8% (431/432)

Electropherograms were base-called with FinchTV and compared using WU-BLAST2 (Altschul et al., 1990) to reference sequences HLA-DRB1*01:01:01 and HLA-DRB1*03:01:01:01 obtained from the IMGT/HLA database (Robinson et al. 2003). Each gap represents a single missing base or an insertion, identities correspond to perfectly matched sequence between reference and sample.

**Table 3:** Electropherograms obtained from sequencing of PCR products following the plasmid enrichment assay

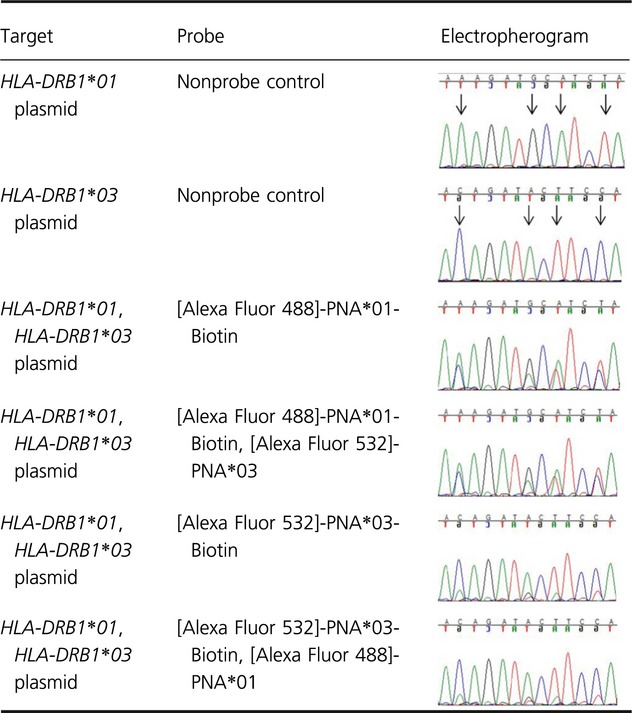

### Haplotyping assay with genomic DNA

**Figure 2:**
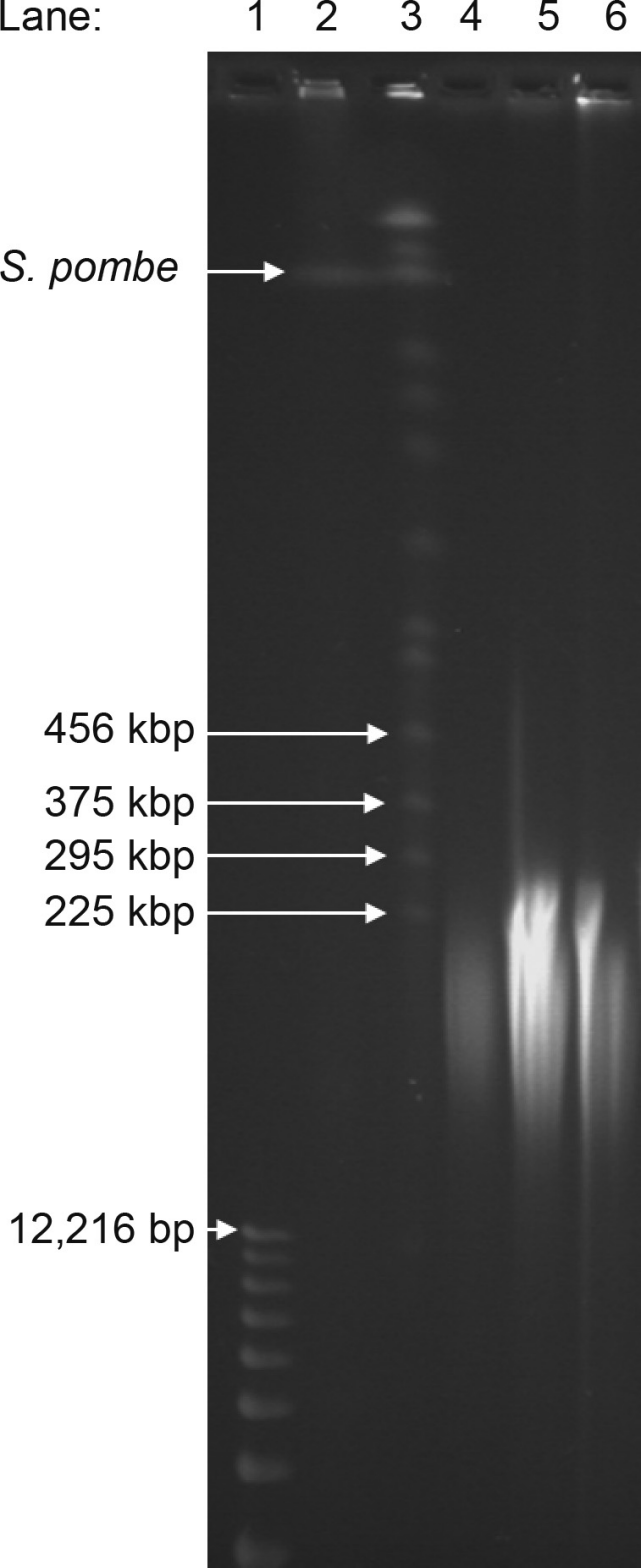
Pulse field gel electrophoresis separation of genomic DNA samples on a 0.8% agarose gel in ×0.5 TBE, at 14°C for 24 h, on a CHEF PFGE System (BioRad), rotating 120° at 6 V/cm, every 60 sec. Lane 1: 1 kb DNA ladder (Invitrogen); Lane 2: *Saccharomyces pombe* DNA ladder; Lane 3: *Saccharomyces cerevisiae* DNA ladder; Lanes 4–6: HLA-DRB1*01,03 heterozygote samples.

Enrichment with high-molecular-weight genomic DNA was performed as per the plasmid enrichment model, using a neutravidin-coated microplate. We selected a microplate-based enrichment approach over magnetic beads in an attempt to minimize any shearing of the high-molecular-weight genomic DNA that could potentially occur during resuspension of the beads during washing. The electropherogram peak heights (Table 3) indicated that the [Alexa Fluor 532]-PNA*03-Biotin probe was the better of the two specific probes at enrichment, so this probe was used for testing the extent of enrichment that could be achieved with high-molecular-weight genomic DNA. High–molecular-weight genomic DNA was purified from a heterozygote individual at HLA-DRB1*01,03 and HLA-DQB1*02,05 (Fig. 2) and probed with [Alexa Fluor 532]-PNA*03-Biotin. Allele-specific quantitation with qPCR for the HLA-DRB1*01 and HLA-DRB1*03 alleles (for probes see Table S1) indicated there was an increased copy number of the HLA-DRB1*03 allele (10,098 copies, S.D. 1567) relative to the HLA-DRB1*01 allele (9181 copies, S.D. 1440), representing an increase in 10.1%, but due to the low number of replicates this result was not statistically significant.

**Figure 3:**
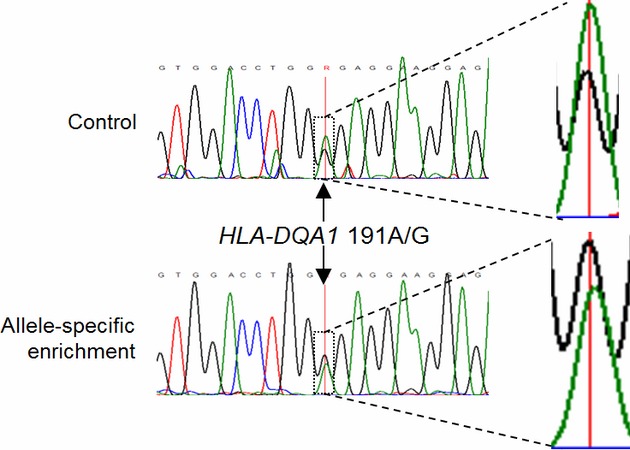
Haplotyping assay performed on high–molecular-weight genomic DNA, viewed with QSV analyzer. The enlarged polymorphic base indicated is a SNP (A>G) at position 191 of the *HLA-DQA1* gene indicates enrichment of the guanine in the HLA-DQA1*05 allele relative to the adenine at the corresponding position in the HLA-DQA1*01 allele. The G>A peak height ratio changed from 0.399 to 0.549, a 37.6% increase in the yield of the HLA-DQA1*05 relative to the HLA-DQA1*01 allele.

To investigate whether enrichment of large fragments of genomic DNA had occurred, the *HLA-DQA1* locus was amplified using generic primers. The *HLA-DQA1* locus is approximately 65 kb from the *HLA-DRB1* locus where HLA-DQA1*05 occurs in strong positive linkage disequilibrium with the HLA-DRB1*03 allele (Louka et al. 2003). Following normalization of the peak heights by adjusting the peak height ratios, the shift in peak heights for SNPs was determined (SNPs were chosen with a minimum of five nonpolymorphic bases flanking the SNP). The peak height ratios shifted 21.7%, 10.8%, and 21.7% and toward enrichment of HLA-DQA1*05 allele at the three polymorphic bases with the first replicate and 37.6% (Fig. 3), 12.5% and 25.8% for the second. The degree of enrichment was estimated by determining the prevalence of different haplotypes of the *HLA-DQA1* gene, which is significantly downstream of *HLA-DRB1*. Sequencing of the PCR products indicated a 99.0% identity match (161/162 nt) with the published HLA-DQA1*05 allele, whereas genomic DNA control samples were only 80.4% matched (131/163 nt). The data indicate that the HLA-DQA1*05 allele was selectively enriched with respect to the HLA-DRB1*01, DQB1*05, DQA1*01 haplotype.

## Discussion

This study demonstrates that specific haplotype DNA can be captured and enriched using biotinylated PNA probes. Strategies for DNA capture predominantly use DNA probes with a biotin-streptavidin enrichment step for use in exome capture and sequencing (Bamshad et al. 2011). Studies aimed at resolving genomic phase resolve either complete chromosome phase (Yang et al. 2011) or assemble phase by fragment reconstruction. Haplotype phasing is useful for whole genome sequencing, pharmacogenomics, complex multigenic disease association (Manolio et al. 2009; Petronis 2010), and preimplantation genetic diagnosis (Handyside et al. 2010). Specific chromosomal fragment phasing (Dapprich et al. 2008) has a role in facilitating the phase sequence information of either highly varied or elaborately organized regions, such as the HLA system.

The feasibility of using PNA probes to enrich DNA samples for both exome sequencing and haplotyping was examined in this study, initially using short target fragments contained in plasmids, and later using samples of genomic DNA from human subjects with known HLA haplotypes. Two alleles of the *HLA-DRB1* gene were targeted to investigate the feasibility of conducting the enrichment of a single allele from a heterozygote. We have shown that two different PNAs could selectively bind to, and facilitate the extraction of plasmid DNA that contained matched sequences to the PNA-binding sequence with a yield of approximately 10 times that of nonprobe, nonbiotinylated and MM probes. Initially, we used the fluorescence of the biotin-PNA probes to confirm that the probe was in solution and was able to bind to neutravidin-coated microplates and confirmed the potential for using biotin-PNAs to selectively purify DNA in mixtures. Despite a relatively large number of washes, there was a consistent amount of nonspecific binding of DNA that did not appear to reflect nonspecific hybridization of PNAs to the DNA. Rather, our results indicated that retention was due to nonspecific interactions of DNA with the microplate or neutravidin-coated surface as the mass of DNA bound in a nonspecific manner was at least as great as the mass bound through specific interactions (Fig. 1). Real-time PCR was used to quantitate the number of target DNA molecules after enrichment experiments. There was a minor but not significant decrease in the apparent yield of the plasmid recovered in experiments performed with the nonbiotinylated PNAs relative to naked DNA controls possibly due to an overlapping binding site of the PNA probes to the PCR forward priming positions. This finding supports the previous indications that PNAs can be used to act as PCR inhibitors (Pellestor and Paulasova 2004), though to a lesser extent than bis-PNAs that are designed to hybridize as a “DNA clamp” (Knudsen and Nielsen 1996). For the plasmid enrichment, the results obtained here were consistent with the degree of specificity indicated in a meta-analysis on PNA/DNA interactions using surface plasmon resonance (Lao et al. 2009). The latter review indicated that PNAs ranging in length between 10 and 22 bases could distinguish PM and MM DNA targets using a range of conditions, though there is a tendency for longer PNAs to require modifications to hybridization conditions to achieve appropriate stringency of binding. A further example of this is described in a published study on PNA/DNA hybridization in 15–20% dimethylformamide at pH 9.5 (Masuko 2003) on a glass slide.

Applying the enrichment strategy to high-molecular-weight genomic DNA, we observed a modest increase of 15–20% enrichment. The degree of enrichment was estimated by determining the prevalence of different haplotypes of the *HLA-DQA1* gene, which is significantly downstream of *HLA-DRB1*. The data indicated that the HLA-DQA1*05:01:01 was selectively enriched with respect to the HLA-DRB1*01-DQB1*05-DQA1*01 haplotype. This is an exciting finding as HLA-DQA1*05:01:01 is in strong positive linkage disequilibrium with the HLA-DRB1*03-HLA-DQB1*02 haplotype (Louka et al. 2003). The DNA extraction method used in this study provided enriched fragments of genomic DNA ranging in length from 50 to 250 kb (Fig. 3). Our data suggest that DNA extraction kits (that readily provide genomic DNA fragments of approximately 10–50 kb in size) can be used for haplotype enrichment and would potentially generate a higher degree of enrichment. Alternatively, in tandem with additional gel-purification of high-molecular-weight genomic DNA, longer fragments of DNA could be haplotyped. In our experiments, DNA was bound to neutravidin-coated plates in a nonspecific manner and this limited the efficiency of allele-specific enrichment due to nonspecific interactions of DNA with the microplate or neutravidin-coated surface discussed above. As the molecular weight of the genomic DNA is between 20 and 60 times that of the plasmid DNA, the PNA ratio to the PM DNA *HLA-DRB1*-binding sites would be equivalently reduced. A reduction in the extent of nonspecific binding could improve the efficiency considerably. During the haplotyping assay on genomic DNA, experiments were also performed with replicates containing 25% and 50% formamide. However, qPCR and sequencing results indicated there was no allelic enrichment (results not shown).

An additional advantage of the technique described in this paper is the retention of the methylation profile of the source DNA as no amplification is performed during enrichment. A role for specific chromosomal fragment enrichment exists where enrichment occurs over genomic distances >100 kb highly varied or elaborately organized regions (Dapprich et al. 2008) as pseudogenes and/or repetitive regions may be incompatible with assembly software, particularly post bisulfite treatment. Modest increases in yield, such as that shown in this study, could be improved by increasing the stringency of the PNA/DNA hybridization conditions and/or increasing the stringency of the microplate well washing. However, the increases indicated herein can be readily applied “as–is” with the support of massively parallel sequencing and appropriate software capable of dealing with subtle fragment proportion differentials (Chiu et al. 2010). This study has indicated that PNAs used for enrichment which are not in the PNA-clamp conformation did not interfere with subsequent PCR amplification. The final and potentially most significant advantage is that as the genomic DNA remains double stranded, this enrichment approach could be combined with library preparation for next-generation sequencing. In coordination with bisulfite sequencing of multiple replicates, this enrichment strategy can resolve haplotypes of one of elaborate regions of the genome, and inherently provide the means of obtaining the corresponding methylation profile. Future directions for this work will aim at improving the specificity of the enrichment and incorporating next-generation sequencing in order to assess the composition of the enriched material.

## Conclusions

This study has demonstrated the feasibility of using biotin-PNA probes to selectively target and enrich alleles of the *HLA-DRB1* gene locus. The *HLA-DRB1* region is complex and a major challenge to analyze. The results indicate that target enrichment of a highly complex region such as HLA can be performed and could be used in combination with genome-wide association studies by next-generation sequencing (Marian 2012). Possible applications include haplotype determination, exome sequencing, assessment of copy number variation, and sequence determination of noncoding genomic regions. Increasing the length of DNA and reduction in the extent of nonspecific binding will be the next stage of development.

## Supporting Information

**Data S1.** Plasmid constructs. **Data S2.** Establishment of conditions for measuring DNA enrichment on neutravidin microplates. **Table S1.** Oligonucleotide primers used for the plasmid generation and sequencing of plasmid enrichment products with qPCR.
