# Supplementary material for: Human leukocyte antigen haplotype phasing by allele-specific enrichment with peptide nucleic acid probes
Source: Mol Genet Genomic Med. 2014 Jan 19;2(3):245–53. doi: 10.1002/mgg3.65 (PMC4049365; doi:10.1002/mgg3.65)
Supplement: Supplementary file 1 [file mgg30002-0245-SD1.doc]

**SUPPORTING MATERIAL**

**Supplementary Method 1 - Plasmid Constructs**

Two plasmids were generated containing 241 bp of either the *HLA-DRB1*01* (GenBank: X88793.1) or the *HLA-DRB1*03* (GenBank: JQ804938.1) second exon sequences, each inserted into the pDONR 201 entry vector (Gateway recombination technology cloning system (Invitrogen)). Homozygote genomic DNA samples were selected as the template DNA to generate PCR fragments using allele-specific primers (Supplementary Table 1). The PCR reaction used 10 μL of 1 x PCR Buffer (Qiagen) with 0.1 – 0.15 μM of forward and reverse allele-specific primers, ~ 100 ng of DNA, 100 μM, 1.5 mM MgCl_2_ and 65.8 μL of dH_2_0. The following cycling conditions were used on a MyCycler Thermal Cycler (Bio-Rad): Cycle 1, 95°C for 5 min (x 1); Cycle 2, 95°C for 30 s, 60°C for 30 s, 72°C for 30 s (x 36); Cycle 3, 72°C for 10 mins. The product size was confirmed on a 1.5 % agarose gel and the reaction was cleaned with a PCR Cleanup Kit (Mobio, Carlsbad, USA). PCR was repeated using the allele-specific Gateway BP primers in place of the standard allele-specific primers (Supplementary Table S1) to generate recombining sequence overhangs. Recombination reactions were undertaken using BP clonase enzyme mix (Invitrogen) as per the manufacturer’s instructions. The resultant plasmids were transformed into competent Stb13 *Escherichia coli* cells by electroporation using a MiniPulser Electroporation System (Bio-Rad). Plasmid DNA was isolated using a HQ Mini Plasmid Purification Kit (Invitrogen) from overnight LB cultures containing 50 mg/mL kanamycin. Products were quantified *via* Nanodrop spectrophotometer and were confirmed as the correct size by running on a 1.5% agarose gel. Products were DNA sequenced (Micromon, Monash University, Australia) and confirmed as *HLA-DRB1*01:01:01* and *HLA-DRB1*03:01:01:01* alleles by comparison with the IMGT/HLA database . Bulk plasmid preparations were made for use in the plasmid enrichment experiments.

**Supplementary Method 2 - Establishment of conditions for measuring DNA enrichment on Neutravidin microplates**

To determine Alexa Fluor 488 fluorescence, a FITC mirror was used with a 490/8 nm excitation and 520/8 nm emission filter, for Alexa Fluor 532 a dual splitting mirror was used with a 530/8nm excitation and 560/10 emission filter. When incubated with the *HLA-DRB1*01* plasmid, the [Alexa Fluor 488]-PNA*01-Biotin probe had a significantly higher level of fluorescence relative to the [Alexa Fluor 488]-PNA*01 probe (P < 0.001; two-way ANOVA). There was no significant difference between the fluorescence of the probe with the *HLA-DRB1*01* plasmid to the *HLA-DRB1*03* plasmid. Similarly, the optimised configuration for detecting the Alexa Fluor 532 flurophore showed that the [Alexa Fluor 532]-PNA*03-Biotin probe significantly increased fluorescence, compared to all other probes (P < 0.001; two-way ANOVA). There was no significant difference between the [Alexa Fluor 532]-PNA*03-Biotin probe irrespective of which of the two plasmid constructs the probe hybridized.

Binding of PNA probes to Neutravidin surfaced microplate was monitored by fluorescence readings from a pre-calibrated Envision 2101 plate-reader. The level of binding (directly proportional to fluorescence) was significantly higher when biotinylated PNAs were present. Following the first wash, fluorescence readings for the biotin labelled probes decreased markedly, indicating most of the probe had been washed off with subsequent washes decreasing in fluorescence ranging from 5.3% to 7.1%.

**Supplementary Table S1**. Oligonucleotide primers used for the plasmid generation and sequencing of plasmid enrichment products with qPCR.

| Primer Name | Sequence 5’ – 3’ | Reference |
| --- | --- | --- |
| *HLA-DRB1*01* Forward | tggcagcttaagtttgaa |  |
| *HLA-DRB1*01* Reverse | gtgtccaccgcggcccgcc |  |
| *HLA-DRB1*03* Forward | ggagtactctacgtctgag |  |
| *HLA-DRB1*03* Reverse | tagttgtccacccggccccgct |  |
| Gateway BP *HLA-DRB1*01* Forward | ggggacaagtttgtacaaaaaagcaggctcgtatctttcttgtggcagcttaagtttgaa |  |
| Gateway BP *HLA-DRB1*01* Reverse | ggggaccactttgtacaagaaagctgggtggtgtccaccgcggcccgcc |  |
| Gateway BP *HLA-DRB1*03* Forward | ggggacaagtttgtacaaaaaagcaggctcgtcgagtttcttggagtactcacgtctgag |  |
| Gateway BP *HLA-DRB1*03* Reverse | ggggacaagtttgtacaaaaaagcaggctctcgatagttgtccacccggccccgct |  |
| Gateway BP Forward (F1) | ggggacaagtttgtacaaaaaagcaggctcg |  |
| Gateway BP Reverse (R2) | ggggaccactttgtacaagaaagctgggtg |  |
| I1-RB1 (*HLA-DRB1*01* Forward) | tcccagtgcccgctccct |  |
| I2-RB2 (*HLA-DRB1*01* Reverse) | acacactcagattctccgctt |  |
| I1-RB9 (*HLA-DRB1*03* Forward) | tggtgggcgttggggcg |  |
| I2-RB28 (*HLA-DRB1*03* Reverse) | acacacacactcagattccca |  |
| GH26 (DQA1 Generic Forward) | gtgctgcaggtgtaaacttgtaccag |  |
| GH27 (DQA1 Generic Reverse) | cacggatccggtagcagcggtagagttg |  |
